# High-repetition-rate ultrafast fiber lasers enabled by BtzBiI_4_: a novel bismuth-based perovskite nonlinear optical material

**DOI:** 10.1515/nanoph-2025-0087

**Published:** 2025-06-30

**Authors:** Xiaohui Du, Chenyue Liu, Zefei Ding, Yuan Zhao, Cunguang Zhu, Yaoyao Wang, Pengpeng Wang

**Affiliations:** 58291 Liaocheng University, Liaocheng, China

**Keywords:** perovskite crystals, saturable absorber, ultrafast fiber lasers, mode-locking, harmonic mode-locking

## Abstract

Recent advances in perovskite crystals have highlighted their exceptional optical properties, making them promising candidates for a wide range of photonic applications. However, the exploration of high-repetition-rate laser systems based on these materials remains underdeveloped, hindering their potential in ultrafast laser technologies and related fields such as optical communications and precision metrology. In this study, we present, for the first time, the saturable absorption characteristics of a novel organic–inorganic hybrid perovskite incorporating the heavy metal bismuth (Bi), specifically N-methylbenzothiazoleBiI_4_ (BtzBiI_4_). The material was integrated as a saturable absorber (SA) into a passively mode-locking erbium-doped fiber laser. By harnessing the exceptional optical nonlinearity of BtzBiI_4_-SA, we successfully achieved stable fundamental mode-locking, harmonic mode-locking, and bound-state soliton mode-locking within a single cavity. The fundamental mode-locking yielded pulses with a duration of 844 fs and a signal-to-noise ratio of 66.15 dB. Additionally, the 142nd-order harmonic solitons attained an impressive repetition rate of 1.3202 GHz. These results represent a significant step forward in the realization of high-repetition-rate fiber lasers utilizing perovskite materials. Our findings highlight the remarkable potential of BtzBiI_4_ as a high-performance nonlinear optical material, paving the way for next-generation ultrafast photonic devices.

## 1 Introduction

High-repetition-rate (HRR) mode-locked fiber lasers have garnered significant attention due to their exceptional performance across diverse fields. For instance, as light sources for optical frequency combs and optical communication systems, HRR fiber lasers can significantly enhance data transmission rates and improve signal integrity [1], [2]. In nanomanufacturing, HRR fiber lasers deliver high-energy-density pulses, enabling precise material processing with minimal thermal damage [3]. Furthermore, in medical imaging and laser surgery, HRR fiber lasers facilitate high-resolution imaging and accurate tissue ablation [4]. To realize HRR pulse fiber lasers, various methods have been explored, including cavity length reduction, the incorporation of comb filters such as Fabry–Perot and micro ring filters, and harmonic mode-locking (HML) techniques, encompassing both active and passive HML [5]. While cavity length plays a crucial role in determining laser repetition rates, its further reduction is constrained by the gain fiber length, rendering it an unsustainable strategy for achieving higher repetition rates [6]. The integration of comb filters alongside highly nonlinear photonic devices within the laser cavity enables HRR generation through dissipative four-wave mixing mode-locking. However, this method is limited by complex fabrication processes for precision filters and significant insertion losses [7]. Active HML achieves high-frequency pulse outputs exceeding 10 GHz, but it is restricted by the slow temporal response of external modulators, leading to relatively broad pulse widths [8]. Conversely, passive HML is regarded as a more favorable approach for achieving HRR, as it represents a specific multipulse operation regime in which pulse peak power is constrained by the soliton area theorem [9]. According to the soliton area theorem, pulse energy is influenced by both dispersion and nonlinear parameters within the laser cavity. In the fundamental mode-locking state, a stable balance between dispersive and nonlinear effects maintains pulse integrity. As pump power increases, this balance progressively deteriorates; when dispersion becomes insufficient to counteract enhanced nonlinearity, the increased pulse energy triggers pulse splitting. Stronger nonlinear effects accelerate this splitting process, leading to an increase in repetition rates and ultimately achieving HRR operation. Eventually, the pulses restabilize due to the combined contributions of dispersion and nonlinearity [10]. In the HML regime, pulses originating from the fundamental mode-locking state undergo splitting and arrange uniformly as pump power increases and polarization states are finely adjusted. The repetition rate of pulses in the HML state is an integer multiple of that in the fundamental mode-locking state, underscoring the role of highly nonlinear photonic devices in enabling HRR generation [11]. Significant advancements in this field have been demonstrated using various saturable absorbers (SAs). In 2012, Grzegorz et al. reported the use of graphene as an SA in an erbium-doped fiber laser (EDFL), achieving a maximum repetition rate of 2.22 GHz (21st-order harmonic) [12]. In 2013, Luo et al. employed a microfiber-based topological insulator Bi_2_Te_3_ in a fiber laser, realizing HML at 2.04 GHz (418th-order harmonic) [13]. Similarly, in 2014, Liu et al. demonstrated mode-locked pulses at 2.5 GHz (369th-order harmonic) using a microfiber-based MoS_2_-SA in an EDFL [14]. More recently, in 2020, Huang et al. utilized MXene (V_2_CT_*x*_) as an SA, successfully achieving 206th-order HML at 1.01 GHz [15]. In 2022, Lee et al. utilized V_2_AlC-based saturable SAs to achieve mode-locked pulses at a repetition rate of 1.3 GHz, corresponding to the 86th-order harmonic, in an EDFL [16]. In the same year, Kong et al. reported the pioneering application of ZnO/Co_3_O_4_ nanocomposites as SAs in EDFLs, generating high-order HML pulses with repetition rates of 1.465 GHz (242nd-order harmonic) and 1.114 GHz (181th-order harmonic) [17]. These studies illustrate the immense potential of advanced materials, including carbon nanotubes, graphene, topological insulators, transition metal dichalcogenides (TMDs), and MXenes, for achieving HRR in ultrafast fiber lasers. These materials exhibit excellent nonlinear optical properties but also face several challenges. For example, graphene, due to its zero bandgap and low optical absorption efficiency, limits its application in high-power lasers [18], [19]. TMDs, although showing tunable bandgap properties and excellent nonlinear absorption performance, typically exhibit direct bandgap characteristics only in their monolayer form, which requires complex synthesis processes [20], [21]. The topological insulator Bi_2_Se_3_, while possessing remarkable topological effects, suffers from low thermal conductivity and optical damage threshold, restricting its stability in high-power laser applications [22], [23], [24]. Although MXene materials exhibit high nonlinear optical responses and ultrafast carrier mobility, their susceptibility to oxidation limits their long-term stability in practical applications [25], [26]. Thus, the search for novel saturable absorber materials, particularly those with enhanced stability, environmental adaptability, and superior nonlinear absorption performance, has become a crucial direction in laser technology research [27], [28], [29], [30], [31]. In this context, we propose a new type of SA – BtzBiI_4_, a bismuth-based organic–inorganic hybrid perovskite with a narrow bandgap of 1.94 eV and a significant nonlinear optical response [32]. Compared to existing materials, BtzBiI_4_ not only exhibits excellent optical absorption properties but also demonstrates improved stability and resistance to oxidation, making it a promising candidate for applications in ultrafast lasers.

Recently, perovskite crystalline materials have demonstrated tremendous potential across diverse high-tech fields due to their exceptional optoelectronic properties and multifunctionality [33], [34]. In photovoltaics, perovskite solar cells have rapidly emerged as formidable alternatives to traditional silicon-based solar cells, owing to their high light absorption coefficients, tunable bandgaps, cost-effective solution processing, and remarkable power conversion efficiencies. Over the past decade, the power conversion efficiencies of perovskite solar cells have increased from an initial 3.8 % to over 25 %, nearing the efficiency limits of single-crystal silicon solar cells [35], [36]. Moreover, the flexibility and transparency of perovskite materials unlock new possibilities for next-generation wearable electronics and building-integrated photovoltaic systems [37], [38]. In light-emitting applications, perovskite light-emitting diodes have gained significant traction in display technologies and lighting devices due to their high color purity, broad color gamut, and exceptional brightness. By engineering the composition and structure of perovskite materials, efficient light emission spanning the ultraviolet to near-infrared regions can be achieved, underscoring their promise for full-color displays and solid-state lighting [39]. Additionally, perovskite materials have exhibited impressive performance in lasers, photodetectors, memory devices, and sensors. Perovskite-based lasers, featuring low lasing thresholds and high gain coefficients, are particularly promising for biomedical imaging and optical communication [40]. Similarly, perovskite photodetectors, renowned for their high sensitivity and broad spectral response, are widely utilized in environmental monitoring and advanced imaging technologies [41]. Furthermore, perovskite-based memories and sensors leverage their excellent electrical and ionic migration properties, offering innovative solutions for high-density data storage and ultra-sensitive detection systems [42], [43]. Collectively, these findings highlight the immense potential of perovskite crystalline materials for applications in photovoltaics, light emission, optoelectronic detection, and storage sensing. Continued advancements in material science and fabrication techniques will undoubtedly further expand the practical utility and performance of perovskite-based devices [44], [45], [46], [47].

In this study, we successfully synthesized lead-free perovskite crystals (BtzBiI_4_) and systematically characterized their morphology, crystal structure, and optical properties. Based on these investigations, the nonlinear optical properties of BtzBiI_4_ nanomaterials were thoroughly explored. A sandwich-structured SA incorporating BtzBiI_4_ nanomaterials was fabricated via a direct coupling method, and its nonlinear absorption behavior at 1.5 μm was evaluated. The carrier dynamics of the material were examined using femtosecond transient absorption spectroscopy, while its nonlinear absorption parameters were further assessed through a balanced twin-detector setup. The fabricated SA demonstrated a modulation depth of 8.51 % and a saturation intensity of 47.19 MW/cm^2^, indicating excellent saturable absorption performance. Additionally, open-aperture Z-scan measurements revealed a third-order nonlinear optical coefficient of 1.03 × 10^−11^ m/W for BtzBiI_4_. Incorporating the BtzBiI_4_-SA into an EDFL cavity enabled the successful realization of stable fundamental mode-locking, high-order HML, and bound-state mode-locking. Specifically, the fundamental mode-locking operation exhibited a repetition rate of 9.294 MHz, a central wavelength of 1,560.388 nm, a 3-dB bandwidth of 3.24 nm, and a pulse duration of 844 fs. Furthermore, the 142nd-order harmonic mode-locking achieved an ultrahigh repetition rate of 1.3202 GHz. The observed bound-state solitons featured a center wavelength of 1,560.04 nm with a pulse separation of 4.073 ps. These results convincingly demonstrate the potential of BtzBiI_4_ as an efficient SA for HRR fiber lasers, paving the way for future developments in ultrafast photonic technologies.

## 2 Synthesis and characterization

### 2.1 Preparation of BtzBiI_4_

**Figure 1:**
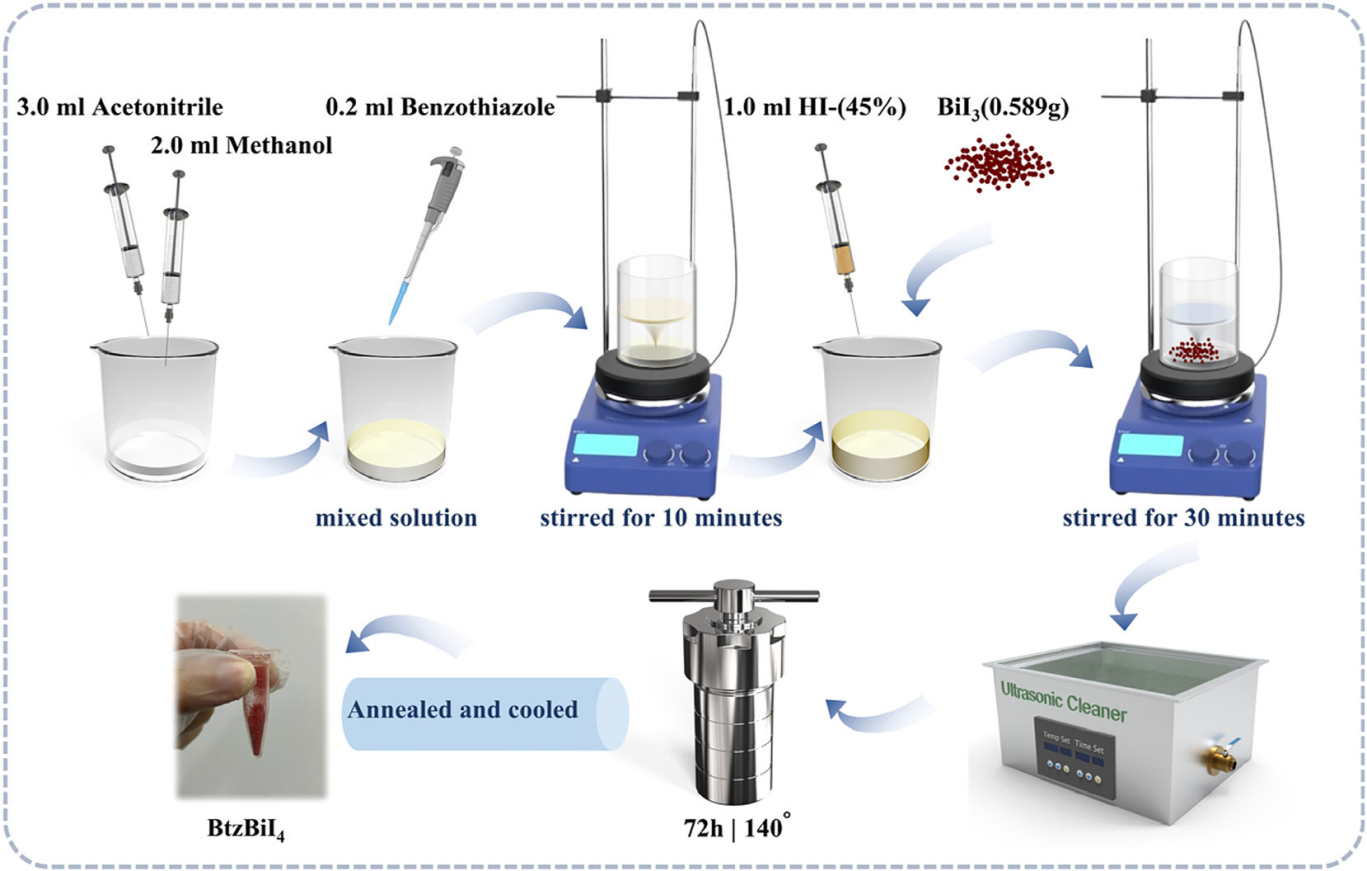
Schematic illustration for the preparation of BtzBiI_4_ crystals.

This research investigates lead-free, bismuth-based perovskite crystals containing ionic liquids, which were successfully synthesized through an *in situ* method. The preparation process, illustrated in Figure 1, involved adding 0.2 mL of benzothiazole to a mixed solution of 3.0 mL acetonitrile and 2.0 mL methanol. The mixture was stirred for 10 min, followed by the addition of 1.0 mL hydriodic acid and 0.589 g BiI_3_. After stirring for 30 min, the resulting red transparent solution was subjected to ultrasonic treatment for 4 h. The solution was then transferred to a 15 mL stainless steel autoclave and heated at 140 °C for 72 h. Gradual cooling to room temperature allowed for the slow formation of high-quality crystals (C_8_H_8_NSBiI_4_, CCDC: 2388135). Upon completion, the product, isolated as a single crystalline phase with an 81 % yield (based on the bismuth content), exhibited distinct red massive crystals and was designated as BtzBiI_4_.

### 2.2 Characterization of BtzBiI_4_

**Figure 2:**
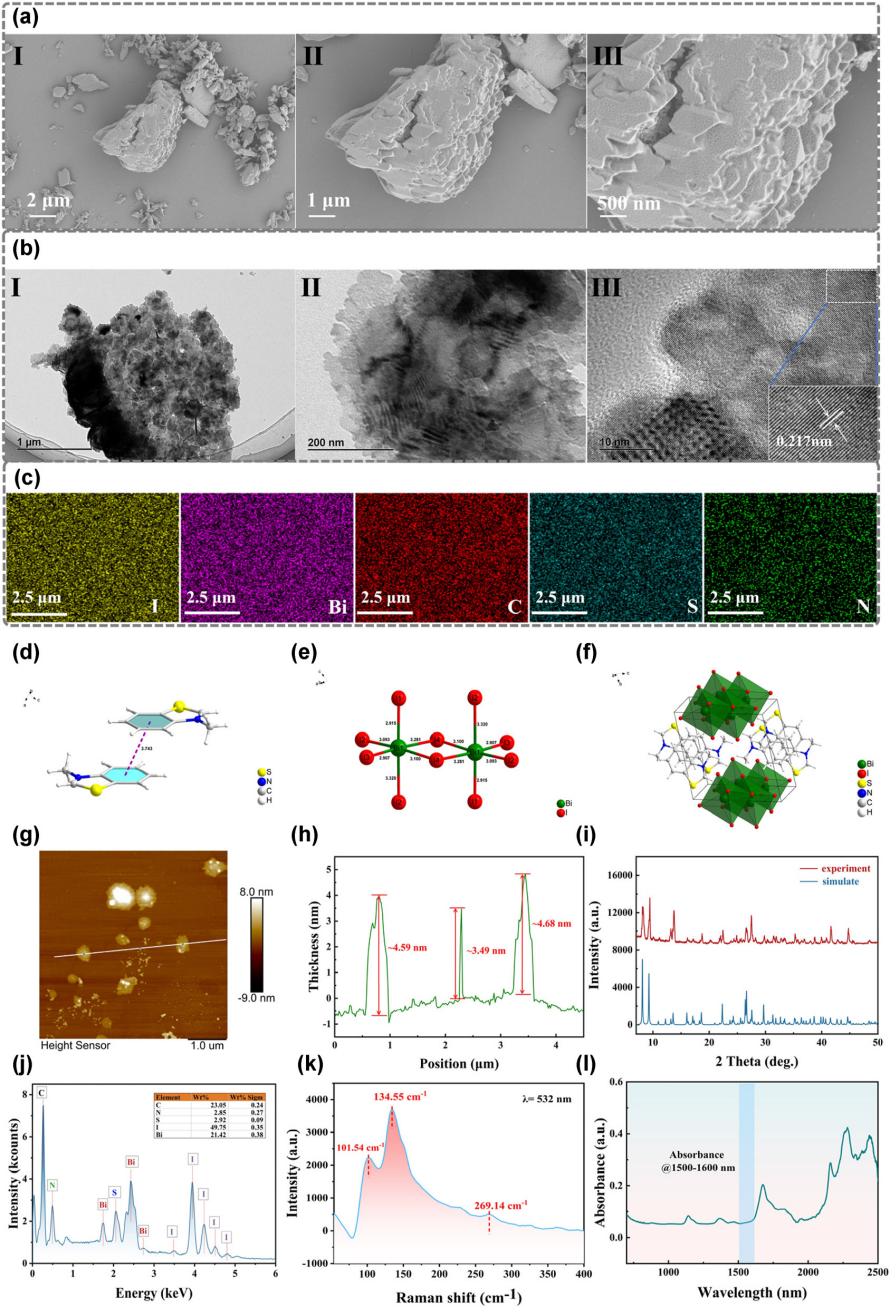
Characterization of BtzBiI_4_. (a) SEM images of BtzBiI_4_, (b) TEM images of BtzBiI_4_, (c) mapping images of BtzBiI_4_, (d) view of the centroid-to-centroid *π*⋯*π* interaction between two adjacent Btz^+^ countercations, (e) the edge shared BiI_6_ octahedral in BtzBiI_4_, (f) side view of single unit cell of BtzBiI_4_ along the ***a*** direction, (g) AFM image of BtzBiI_4_ nanosheets, (h) height profile extracted from the AFM image, illustrating the thickness characteristics, (i) XRD pattern of BtzBiI_4_ powder, (j) EDX spectrum of BtzBiI_4_ powder, (k) Raman spectrum of BtzBiI_4_ powder, and (l) UV-VIS-NIR spectrum of BtzBiI_4_ powder.

The morphology, crystal structure, optical absorption, and scattering properties of the synthesized BtzBiI_4_ crystals were characterized prior to use. After coating the sample with gold for 1 min using an ion sputtering apparatus (GVC-2000, Oxford Quorum), the morphology and particle size distribution of the BtzBiI_4_ dispersion were examined using a scanning electron microscope (SEM, Zeiss Sigma 300). Figure 2a(I)–(III) show images at scales of 2 μm, 1 μm, and 500 nm, respectively. The dispersed material exhibited an irregular block-like morphology (Figure 2(a)) with clear layered structures visible in Figure 2a(I) and (II). Transmission electron microscopy (TEM, FEI Tecnai F20) was used to analyze the crystal distribution at micro- and nanoscale levels. Figure 2b(I)–(III) show images at scales of 1 μm, 200 nm, and 10 nm, respectively. The BtzBiI_4_ samples displayed uniform lattice arrangements with consistent distribution. A selected lattice fringe in Figure 2b(III) reveals a calculated lattice spacing of 0.217 nm, indicating high crystallinity and structural stability of BtzBiI_4_. Elemental distribution was analyzed using energy-dispersive spectroscopy (EDX, Oxford Xplore 30), and the EDX mapping results are shown in Figure 2(j). The analysis indicated a mass ratio of Bi to I of approximately 2:5, consistent with the molecular formula. Figure 2(c) presents the elemental mapping, where yellow, purple, red, blue, and green represent I, Bi, C, S, and N, respectively, confirming the uniform distribution without agglomeration. Single-crystal X-ray diffraction (SCXRD) analysis reveals that the BtzBiI_4_ molecular structure consists of two primary components: a discrete M^+^ cation (with M corresponding to Btz in BtzBiI_4_) and a one-dimensional, continuous anionic chain of (BiI_4_)^−^ (Figure 2(d) and (e)). Within the unit cell of BtzBiI_4_ (Figure 2(f)), eight (Btz)^+^ cations are symmetrically arranged along the four sides of the unit cell, whereas the (Bi_2_I_10_)^4−^ anionic fragments are located at the base-centered positions.

To precisely determine the thickness of the BtzBiI_4_ nanosheets employed in the sandwich-structured SA, atomic force microscopy (AFM) analysis was carried out using a Bruker Dimension ICON system. As presented in Figure 2(g), the AFM image reveals that the nanosheets exhibit a uniform and smooth surface morphology, indicative of high-quality material preparation. The corresponding height profile shown in Figure 2(h) confirms that the nanosheets possess a thickness ranging from approximately 3–5 nm. This ultrathin configuration significantly enhances the light–matter interaction at the nanoscale, thereby contributing to efficient nonlinear optical modulation, which is crucial for the realization of high-performance ultrafast photonic devices. The crystal structure of BtzBiI_4_ was examined via X-ray diffraction (XRD) using a Bruker D8 Advance system. As depicted in Figure 2(i), the blue and red lines represent the simulated and experimental values, respectively. The experimental diffraction spectrum exhibits pronounced peaks at 2*θ* = 8.18°, 9.34°, 13.67°, 27.46°, and 41.66°, which align precisely with the simulated diffraction peaks. To further investigate the molecular structure of BtzBiI_4_, Raman spectroscopy (Horiba Scientific Labram HR Evolution) was employed, and the Raman scattering spectra are shown in Figure 2(k). Three characteristic Raman vibrational modes were identified at 101.54 cm^−1^, 134.55 cm^−1^, and 269.14 cm^−1^, corresponding to the Bi–I bonding structure within the (BiI_4_)^-^ unit. These results are consistent with those reported for (BAH)BiI_4_ crystals in previous studies [48]. To elucidate the absorption properties of BtzBiI_4_ nanosheets, Ultraviolet-Visible-Near Infrared (UV-VIS-NIR) optical spectra were measured using a UV-3600-Plus spectrophotometer (Shimadzu). The absorption spectra, as shown in Figure 2(l), exhibit broadband absorption in the 1,500–1,600 nm range. Overall, these characterization results demonstrate that BtzBiI_4_ possesses remarkable crystallinity, uniform particle size distribution, and absorption capability at 1.5 µm, thereby ensuring its reliability and stability for optoelectronic device applications.

## 3 Nonlinear optical response

### 3.1 Fabrication of BtzBiI_4_-SA

**Figure 3:**
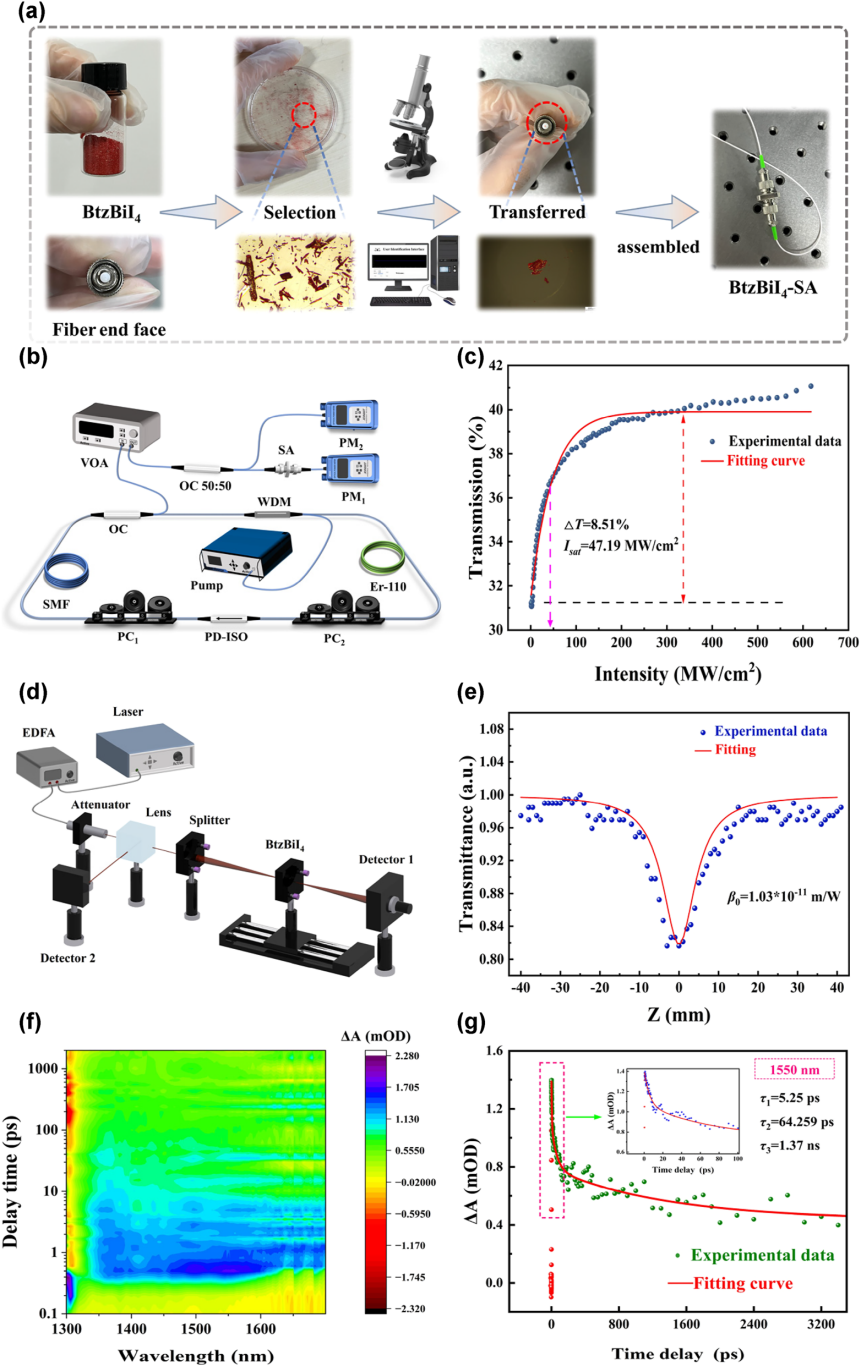
Characterization of BtzBiI_4_. (a) Preparation of BtzBiI4-SA. (b) Schematic of the balanced twin-detector technique. (c) Saturable absorption characteristics of BtzBiI4-SA. (d) Schematic diagram of open-aperture Z-scan system. (e) Z-scan curves of BtzBiI4 nanomaterials. (f) The 2D transient absorption color-coded map of BtzBiI4 material. (g) Dynamic curves at 1,550 nm of BtzBiI4 nanomaterials.

A sandwich-structured BtzBiI_4_-SA was successfully fabricated using a direct coupling technique, and its nonlinear saturable absorption properties were systematically characterized. To prepare the device, 10 mg of the synthesized BtzBiI_4_ crystals were placed in a glass petri dish for selection. Using an optical microscope (DVE3630, Otter Optics), a crystal with a relatively regular morphology and a diameter of approximately 125 μm was identified. The selected crystal was then carefully transferred onto the end face of an optical fiber using a fiber probe. Under microscopic observation, it was confirmed that the crystal completely covered the fiber core, ensuring effective coupling. Finally, two optical fiber jumpers and a flange were assembled to create the sandwich structure, thereby completing the fabrication of the BtzBiI_4_-SA (Figure 3(a)).

### 3.2 Saturable absorption of BtzBiI_4_-SA

Examining the nonlinear optical response, underlying mechanisms, and the interaction of light with nanostructures provides a broader context for understanding the unique characteristics of BtzBiI_4_-SA. Recent studies on the nonlinear behavior of quantum dot materials and the interaction of light with nanostructures offer valuable comparisons to our work. For instance, Yu et al. [49] investigated the nonlinear optical response of lead telluride quantum dots in all-solid-state pulse lasers, while Zhu et al. [50] developed MXene V_2_CT_x_ nanosheet/bismuth quantum dot heterostructures, enhancing nonlinear optical properties. Additionally, Finke et al. [51] explored temperature-resistant quantum dot saturable absorbers for integration into semiconductor lasers. Other studies, including those on Ag_2_S quantum dots [52] and phosphorene quantum dots [53], focus on their applications in biosensing and optoelectronic detection [54], [55]. Building on these insights, the nonlinear optical response of BtzBiI_4_-SA was systematically investigated using a balanced twin-detector configuration, as shown in Figure 3(b). This approach provides a detailed understanding of the material’s nonlinear characteristics, essential for its potential in ultrafast photonic applications. The experimental apparatus comprises a high-power seed laser, a tunable variable optical attenuator (pVOA-1550-1-B-30-SM-B, Max-ray Photonics), and an optical coupler (OC). The seed laser, a custom-built erbium-doped fiber laser employing nonlinear polarization rotation (NPR) for mode-locking, generates pulses at a central wavelength of 1,562.32 nm, with a pulse width of 788 fs and a repetition frequency of 25.12 MHz. The NPR fiber laser system integrates a 976 nm laser diode (Model: PUMPL-976-1550-FA-B-YP), two polarization controllers (PCs), and a polarization-dependent isolator (PD-ISO) to sustain stable NPR mode-locking. Additional components include erbium-doped fiber (EDF), single-mode fiber (SMF-28), a wavelength division multiplexer (WDM), and an optical coupler. In this configuration, 20 % of the laser output is guided through the tunable attenuator and subsequently divided into two channels by a 50:50 optical coupler. One path is directly injected into a power meter, while the other path passes through the BtzBiI_4_-SA before being directed into the same power meter. By adjusting the parameters of the optical attenuator, the output power of the NPR fiber laser can be modulated. A nonlinear transmission curve was derived from the measured data and subsequently fitted using a standard saturable absorption model (Eq. (1)).

(1) $$T\left(I\right)=1-\Delta T\exp\left(-I/I_{\text{sat}}\right)-T_{\text{ns}}$$

In the nonlinear absorption model, *T*(*I*) represents the transmittance, Δ*T* is the modulation depth, *I* is the incident light intensity, *I*_sat_ is the saturation intensity, and *T*_ns_ denotes the nonsaturable losses. The measurement results are shown in Figure 3(c), indicating that as the incident light power increases, the absorption of BtzBiI_4_-SA gradually decreases and eventually reaches saturation. The Δ*T* is 8.51 %, the *I*_sat_ is 47.19 MW/cm^2^, and the *T*_ns_ is 60.09 %. These results demonstrate that BtzBiI_4_-SA exhibits excellent nonlinear saturable absorption properties, making it a promising SA for passive mode-locking fiber lasers. Compared with conventional SAs (Table S1, see Supplementary Material), BtzBiI_4_ exhibits a MD of 8.51 %, which is competitive with materials such as NbTe_2_ and BP. Notably, BtzBiI_4_ demonstrates an exceptionally high *I*_sat_ of 47.19 MW/cm^2^, surpassing many reported materials (e.g., MXene-film and MXene-DS), and thus shows great potential for high-power ultrafast laser applications. A high *I*_sat_ typically indicates strong tolerance to intense laser irradiation, although it may also imply an elevated damage threshold. For ultra-high-power lasers, appropriate protective measures are recommended to ensure material stability and prevent damage under prolonged high-intensity exposure. Overall, BtzBiI_4_ presents significant advantages as a saturable absorber, especially for high-power and HRR lasers. Nevertheless, for certain extreme operating conditions, it is crucial to balance the high saturation intensity with the material’s damage threshold to ensure long-term operational reliability.

The saturable absorption properties of 2D materials are fundamental to their role as absorbers in pulsed laser systems. At low light intensities, these materials exhibit linear absorption. However, as the incident optical power increases, their absorption behavior becomes nonlinear. As the intensity rises, the absorption decreases, and the transmission increases, which is characteristic of the saturable absorption effect in 2D materials [56]. To probe the nonlinear absorption characteristics of BtzBiI_4_, the open-aperture Z-scan technique, based on spatial beam distortion, was employed for the measurements. As illustrated in Figure 3(d), a custom-built NPR mode-locking fiber laser, amplified by an erbium-doped fiber amplifier (EDFA-PA35-B), was used as the light source for the Z-scan system. The optical beam was collimated using a fiber collimator (GCX-L30APC-1550) and subsequently split by a 10:90 beam splitter (GCC-403234). Ninety percent of the beam was directed through a focusing lens with a focal length of 50.8 mm (GCL-M010109N) and a quartz plate (*φ*25 mm × 2  mm, infrared, JGS3) coated with BtzBiI_4_ material, and then detected by a thermopile detector (GCI-080202). The power was recorded using an optical power meter (GCI-08). The remaining 10 % of the beam was directly detected by a second thermopile detector as a reference. During the experiment, the power values were varied by continuously adjusting the position of the quartz plate along a sliding track. The *Z*-axis zero point was positioned at the focal point of the lens, with the propagation direction of the beam aligned along the positive *Z*-axis. As the sample moved along the positive *Z*-axis, the intensity of the incident light varied due to the self-focusing or self-defocusing effects occurring within the sample. By comparing the data recorded by detector 1 and detector 2, the nonlinear absorption properties of the sample were obtained. The normalized transmittance *T*(*z*) was calculated using the following formula (Eq. (2)):

(2) $$T\left(z\right)=1-\beta_{0}\cdot L_{\text{eff}}\cdot I_{0}/\left[2\sqrt{2}\left(1+z^{2}/z_{0}^{2}\right)\right]$$

where *T* is the normalized transmittance, *z* represents the relative position of the sample along the *Z*-axis with respect to the lens focal point, *L*_eff_ denotes the effective length of the material, *z*_0_ is the Rayleigh length, and *β*_0_ and *I*_0_ correspond to the nonlinear absorption coefficient and the peak axial intensity at the focal point (*z* = 0), respectively. As shown in Figure 3(e), the *β*_0_ was determined to be 1.03 × 10^−11^ m/W through curve fitting. Nonlinear absorption typically results from a combination of SA, reverse saturable absorption, and two-photon absorption. While reverse saturable absorption and two-photon absorption contribute to enhanced optical absorption, saturable absorption leads to an increased irradiance threshold for effective absorption in the material. This interplay of mechanisms defines the material’s nonlinear optical behavior and its potential applications in ultrafast photonic devices.

### 3.3 Transient absorption characteristics of the as-prepared BtzBiI_4_ nanomaterials

To explore the ultrafast carrier dynamics of BtzBiI_4_ in greater detail, femtosecond time-resolved transient absorption (TA) spectroscopy was applied to investigate the carrier relaxation pathways (Figure S1; see Supplementary Material). An 1,150 nm pump laser was utilized to promote electrons from the ground state to the excited state, while a broadband white light source spanning 1,300–1,700 nm functioned as the probe beam. The delay time, representing the temporal difference between the pump and probe pulses, was employed to monitor the evolution of the carrier dynamics. The TA modulation signals (Δ*A* = *A*_1_ − *A*_0_) were derived by measuring the absorption levels in the presence (*A*_1_) and absence (*A*_0_) of pump excitation, providing insights into the absorption behavior under high and low excitation conditions. As illustrated in Figure 3(f), BtzBiI_4_ exhibits broadband absorption near 1,550 nm, with pronounced absorption in the long-wavelength region (>1,300 nm) facilitated by electronic transitions. Figure 3(g) presents the dynamic curve at a probe wavelength of 1,550 nm, showing the evolution of Δ*A* with delay time. These results demonstrate a dynamic transition from photoinduced excited-state absorption to a bleaching signal. The TA curves were fitted using a three-exponential decay function (Eq. (3)):

(3) $$\begin{array}{ll} \Delta A & =A_{1}\cdot \exp\left(-t/\tau_{1}\right)+A_{2}\cdot \exp\left(-t/\tau_{2}\right)\\ & \;+A_{3}\cdot \exp\left(-t/\tau_{3}\right) \end{array}$$

In this context, *A*_1_, *A*_2_, and *A*_3_ denote the amplitude components, while *t* represents the delay time. The parameters *τ*_1_, *τ*_2_, and *τ*_3_ correspond to the response times associated with distinct carrier dynamic processes. Upon pump excitation, ground-state electrons – including those localized at oxygen vacancy defects – are elevated to excited states. When subjected to probing at different wavelengths, these excited electrons absorb photon energy, facilitating their transition to higher excited states. This phenomenon manifests as photoinduced absorption peaks, which are indicated by the dashed regions in Figure 3(g). The formation of photoinduced absorption peaks is closely associated with carrier thermalization and relaxation processes [57]. The high intensity of the photoinduced absorption peaks indicates the strong photo absorption capability and high carrier density of BtzBiI_4_, underscoring its excellent saturable absorption properties and significant potential for nonlinear optical applications. Moreover, the rapid picosecond-scale decay process is attributed to bulk electron–hole recombination, while the longer bleaching duration is linked to defect-state electron trapping and recombination with holes [58], [59], [60]. The transient absorption characteristics of BtzBiI_4_ are particularly vital for mode-locking lasers. A shorter *τ*_1_ facilitates rapid responses, enabling the generation of ultrashort pulses in mode-locking lasers. Meanwhile, intermediate *τ*_2_ and longer *τ*_3_ contribute to stabilized mode-locking and HRR pulse outputs, making BtzBiI_4_ highly suitable for applications requiring pulse-width tuning or pulse shaping.

## 4 Experimental setup

**Figure 4:**
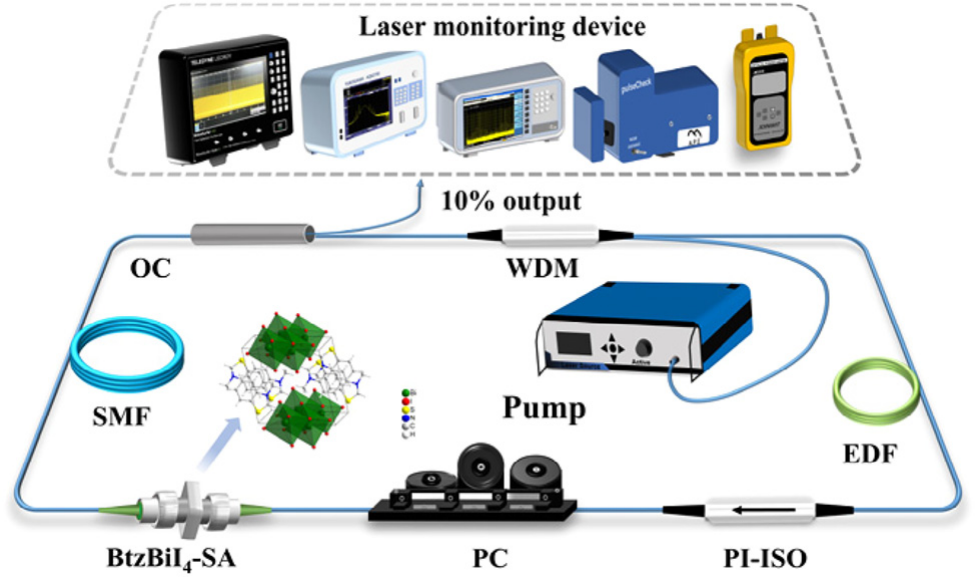
Schematic of the erbium-doped fiber laser.

The schematic of the ring-cavity passively mode-locking EDFL employing the BtzBiI_4_-SA is illustrated in Figure 4. The system is pumped by a 976 nm laser diode (LD PUMPL-976-1550-FA-B-YP), with light coupled into the ring cavity through an integrated optical module that includes a 976/1,550 nm WDM, a polarization-independent isolator (PI–ISO), and a 10/90 OC. The PI-ISO enforces unidirectional light propagation within the cavity, preventing potential damage from back reflections. The OC directs 10 % of the intracavity power as output, with the remaining 90 % recycled to sustain oscillation. A PC fine-tunes the polarization state and adjusts cavity birefringence. The gain medium, a 32-cm EDF (Er-110 Nufern SM-ESF-4/125), exhibits a dispersion of −46 ps/nm/km at 1,550 nm. Nonlinear phase shifts are accumulated through 12 m of SMF (G652D SMF-28, Corning) with a dispersion of 17 ps/nm/km at 1,550 nm. Accounting for the pigtail lengths of all components, the total cavity length measures 22.11 m, corresponding to a net dispersion of −0.4534 ps^2^. The BtzBiI_4_-SA, serving as the mode-locking element, demonstrates excellent saturable absorption properties by transmitting high-intensity components of the stimulated emission while attenuating weaker ones. This iterative process enables the formation of a stable single pulse after thousands of cavity round trips. For pulse characterization, the system includes a spectrum analyzer (AQ6370D, Yokogawa), a digital oscilloscope (MDO3102, Tektronix), a radio frequency spectrum analyzer (FPC1000, Rohde & Schwarz), an autocorrelator (PulseCheck 50, APE), a 3 GHz photodetector (PD-03-FA-D, Max-ray Photonics), and an optical power meter (JW3208C, Joinwit).

## 5 Results and discussion

### 5.1 Fundamental mode locking

**Figure 5:**
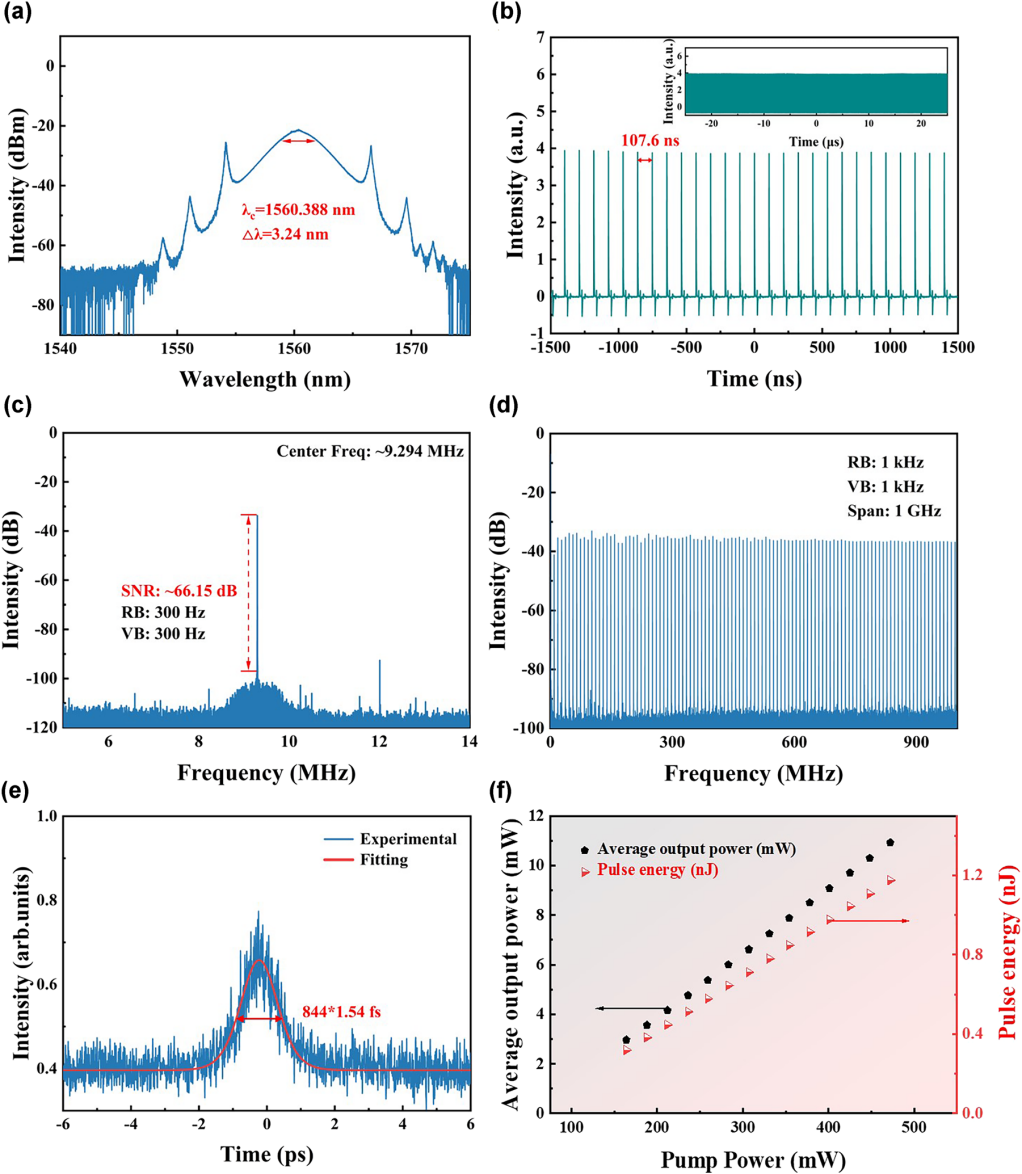
Characteristics of fundamental mode locking. (a) Optical spectrum. (b) Oscilloscope trace. (c) RF spectrum. (d) RF spectrum within the 1 GHz range. (e) Autocorrelation trace. (f) Spectral variations at different pump powers.

When the coupled pump power was increased to 97 mW, mode-locking was initiated, leading to the generation of multiple pulses within the cavity. By adjusting the polarization state, a pulse train with a repetition rate of 9.294 MHz was achieved. Under the fundamental mode-locking state, the laser’s performance was evaluated at an output power of 365 mW, as recorded in Figure 5. To begin with, the mode-locking spectrum reveals a central wavelength of 1,560.388 nm, accompanied by a 3 dB spectral bandwidth of 3.24 nm (Figure 5(a)). Notably, the presence of distinct Kelly sidebands in the spectrum confirms that the laser operates within the conventional soliton regime, signifying soliton-like behavior. Furthermore, the pulse train shown in Figure 5(b) exhibits uniformly spaced pulses, which clearly indicate stable mode-locking operation. The pulse interval, measured as 107.6 ns, corresponds precisely to the cavity length of 22.11 m, thereby validating the experimental setup’s precision. In addition, a detailed view of a 5 μs pulse sequence highlights consistent spacing and stable intensity, further emphasizing the reliability and temporal stability of the mode-locking mechanism. Moving on to the pulse duration, the autocorrelation trace fitted with a sech^2^ function reveals a duration of 844 fs (Figure 5(c)). The calculated time-bandwidth product (TBP) is 0.3529, close to the Fourier transform limitation (0.315) for sech^2^-shaped pulses, suggesting the presence of slightly chirped pulses. To further assess the laser’s operational stability, the radio frequency (RF) spectrum was analyzed (Figure 5(d)). The measured signal-to-noise ratio (SNR) of 66.15 dB underscores the high stability of the BtzBiI_4_-SA-based laser system. Additionally, the pulse spectrum recorded over a 1 GHz span confirms uniformly distributed pulses, providing further evidence of excellent stability and pulse quality (Figure 5(e)). As the pump power increased from 164 mW to 472 mW, both the average output power and single-pulse energy exhibited a linear growth trend. The average output power rose from 2.97 mW to 10.92 mW, while the corresponding single-pulse energy increased from 0.319 nJ to 1.175 nJ (Figure 5(f)). This linear relationship suggests efficient energy conversion in the laser cavity, reflecting the stability and scalability of the mode-locking performance under varying pump powers. The optical spectra at various power levels, the 12-h pulse train at fixed pump power, and the 12-h output power stability test indicate that the fabricated SA maintains long-term stable operation under high irradiance without degradation, ensuring consistent and high-quality laser output (Figure S2a–c; see Supplementary Material).

### 5.2 Harmonic mode locking

**Figure 6:**
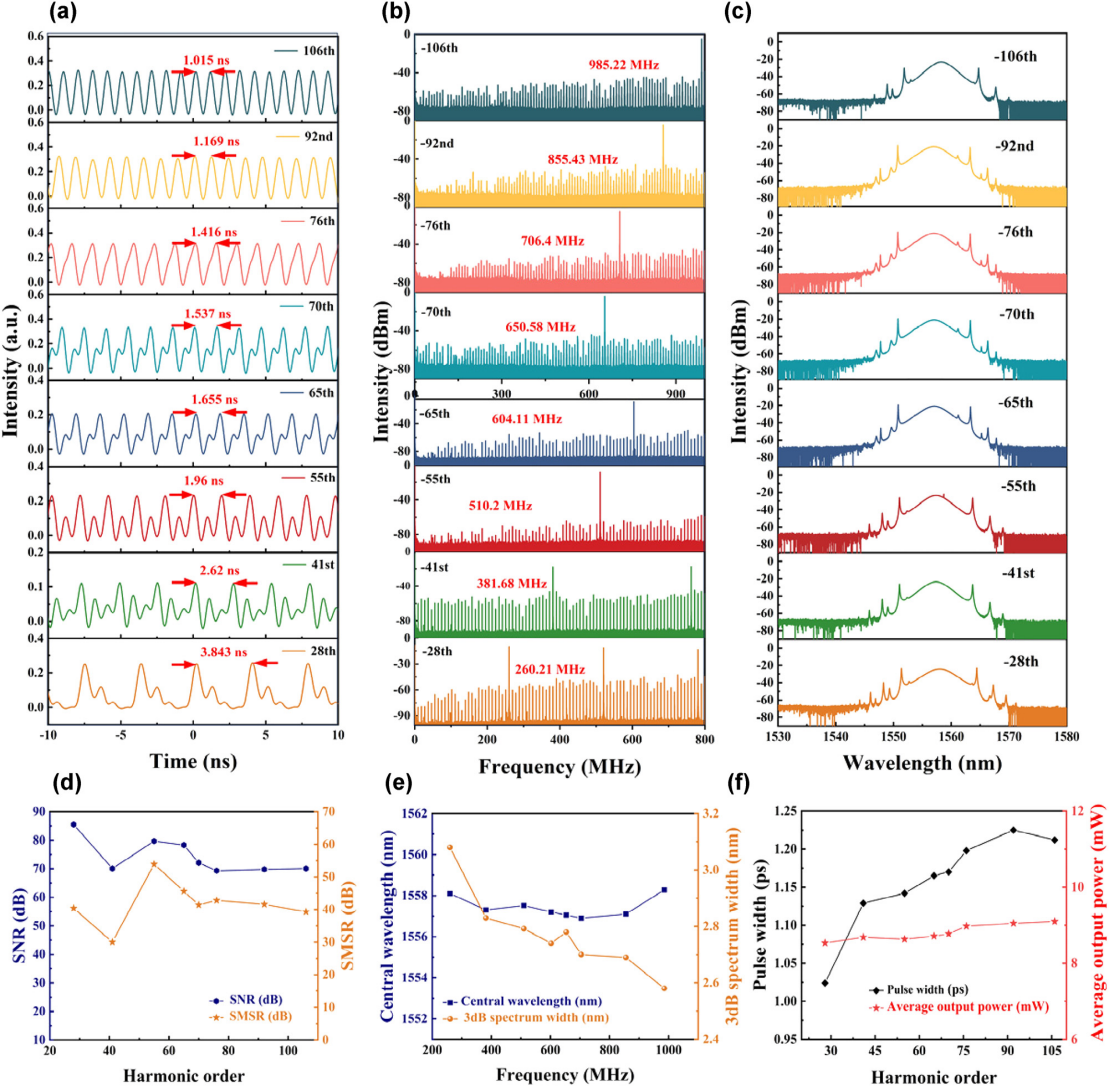
Harmonic soliton output characteristics based on BtzBiI_4_-SA: (a) oscilloscope trace. (b) RF spectrum. (c) Optical spectrum. (d) SNR and SMSR of harmonic mode-locking pulses. (e) Central wavelength and 3 dB bandwidth variation with repetition frequency. (f) Pulse width and average output power variation with harmonic order.

By carefully tuning the PC and progressively increasing the pump power, HML pulses with varying repetition rates were successfully generated. During the transition to higher harmonic orders, transient instabilities in the pulse train were observed, which subsequently stabilized into new harmonic states as a result of intracavity nonlinear effects and gain competition. At a pump power of 391 mW, the pulse characteristics under the 28th, 41st, 55th, 65th, 70th, 76th, 92nd, and 106th HML conditions are presented in Figure 6. As shown in Figure 6(a), the pulse trains of different harmonic orders exhibit uniform and stable distributions. The measured peak time intervals were 3.843 ns, 2.62 ns, 1.96 ns, 1.655 ns, 1.537 ns, 1.416 ns, 1.169 ns, and 1.015 ns, respectively. These intervals directly correspond to the repetition frequencies of the harmonic pulses, demonstrating a significant decrease in pulse spacing as the harmonic order increases. The RF spectra in Figure 6(b) validate the precise repetition frequencies, measured as 260.21 MHz, 381.68 MHz, 510.2 MHz, 604.11 MHz, 650.58 MHz, 704.23 MHz, 855.43 MHz, and 985.22 MHz for the respective harmonic orders. Furthermore, the SNRs of these pulses exceed 70 dB, indicating stable signals with minimal noise, while the side-mode suppression ratios (SMSRs), all above 30 dB, confirm excellent spectral purity and reduced mode competition (Figure 6(d)). The optical spectra of the HML pulses, depicted in Figure 6(c), exhibit typical soliton features with prominent Kelly sidebands. The central wavelength remains stable around 1,558 nm, as illustrated in Figure 6(e). However, the 3 dB spectral bandwidth narrows from 3.08 nm to 2.58 nm as the harmonic order increases, likely due to spectral filtering effects in high-order harmonics. The relationship between harmonic order, pulse duration, and average output power is detailed in Figure 6(f). Notably, while the pulse duration broadens from 1 ps to 1.25 ps with increasing harmonic order—likely due to nonlinear effects, dispersion, and phase-matching constraints—the average output power remains nearly constant, indicating conserved energy per pulse (Figure S3a–h; see Supplementary Material).

**Figure 7:**
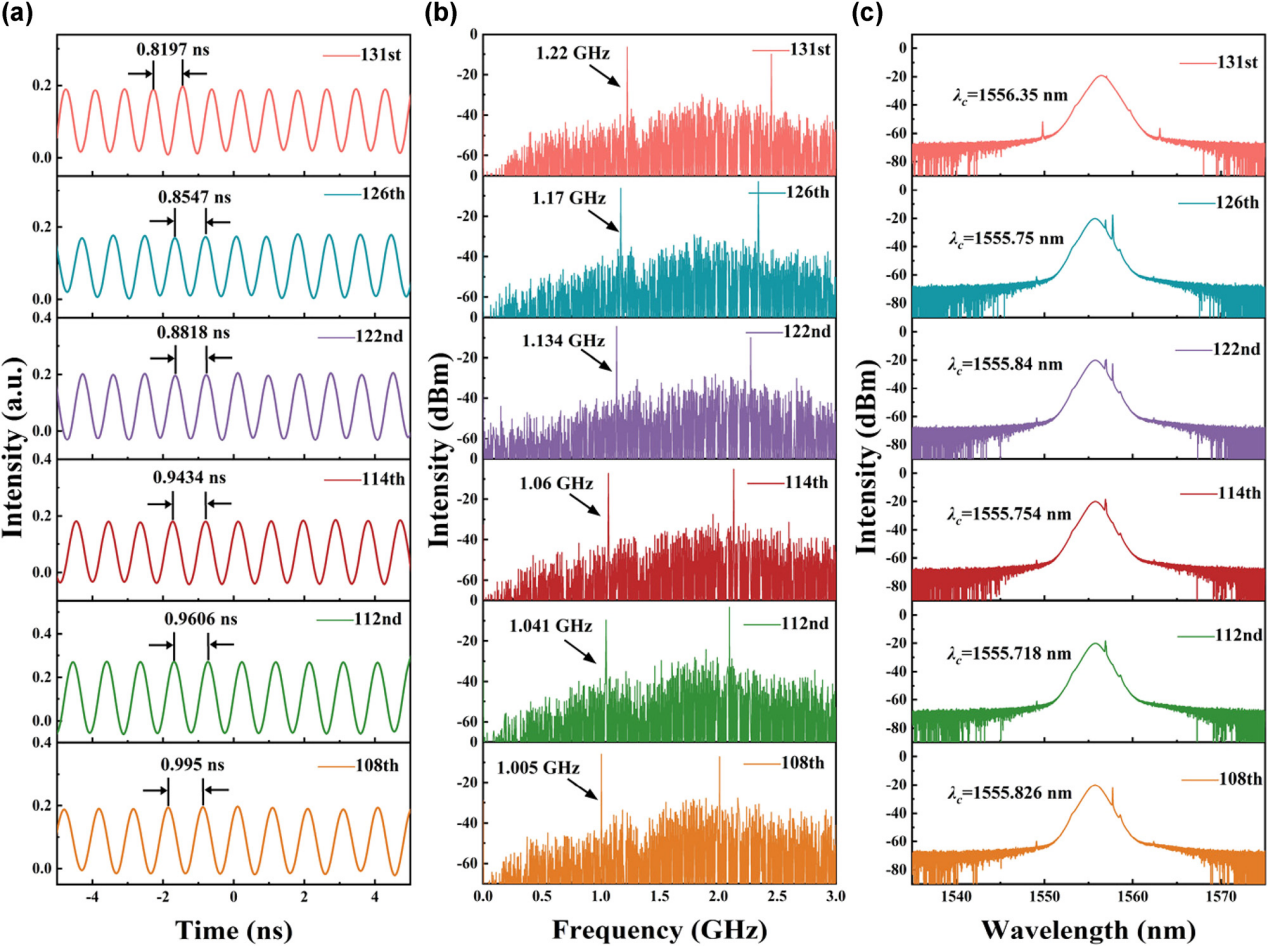
Harmonic soliton output characteristics based on BtzBiI_4_-SA: (a) oscilloscope trace. (b) RF spectrum. (c) Optical spectrum.

By gradually increasing the pump power, the repetition rate exceeded 1 GHz when the pump power was adjusted to 413 mW. Further tuning of the polarization state enabled the capture of HML at the 108th, 112nd, 114th, 122nd, 126th, and 131th orders. Detailed pulse characteristics are presented in Figure 7. Figure 7(a) shows the pulse train captured by the oscilloscope, where the pulse intervals correspond to 1/108, 1/112, 1/114, 1/122, 1/126, and 1/131 of the fundamental mode-locking pulse interval. The exact repetition frequencies were measured by a radio frequency spectrum analyzer (DSA875, RIGOL) with a resolution of 3 kHz (Figure 7(b)), yielding values of 1.005 GHz, 1.041 GHz, 1.06 GHz, 1.134 GHz, 1.17 GHz, and 1.22 GHz. In all cases, the SMSRs exceeded 30 dB, demonstrating the stability of harmonic mode-locking. The spectral profiles remained consistent across different harmonic orders. As the pump power continued to increase, enhanced nonlinear effects within the cavity led to significant suppression of the Kelly sidebands. The central wavelength stabilized around 1,556 nm, with a 3 dB bandwidth of approximately 1.6 nm (Figure 7(c)). Furthermore, autocorrelation measurements indicated pulse durations ranging from 1.49 ps to 1.75 ps, confirming high-quality pulse characteristics in the HML regime (Figure S4a–f; see Supplementary Material).

**Figure 8:**
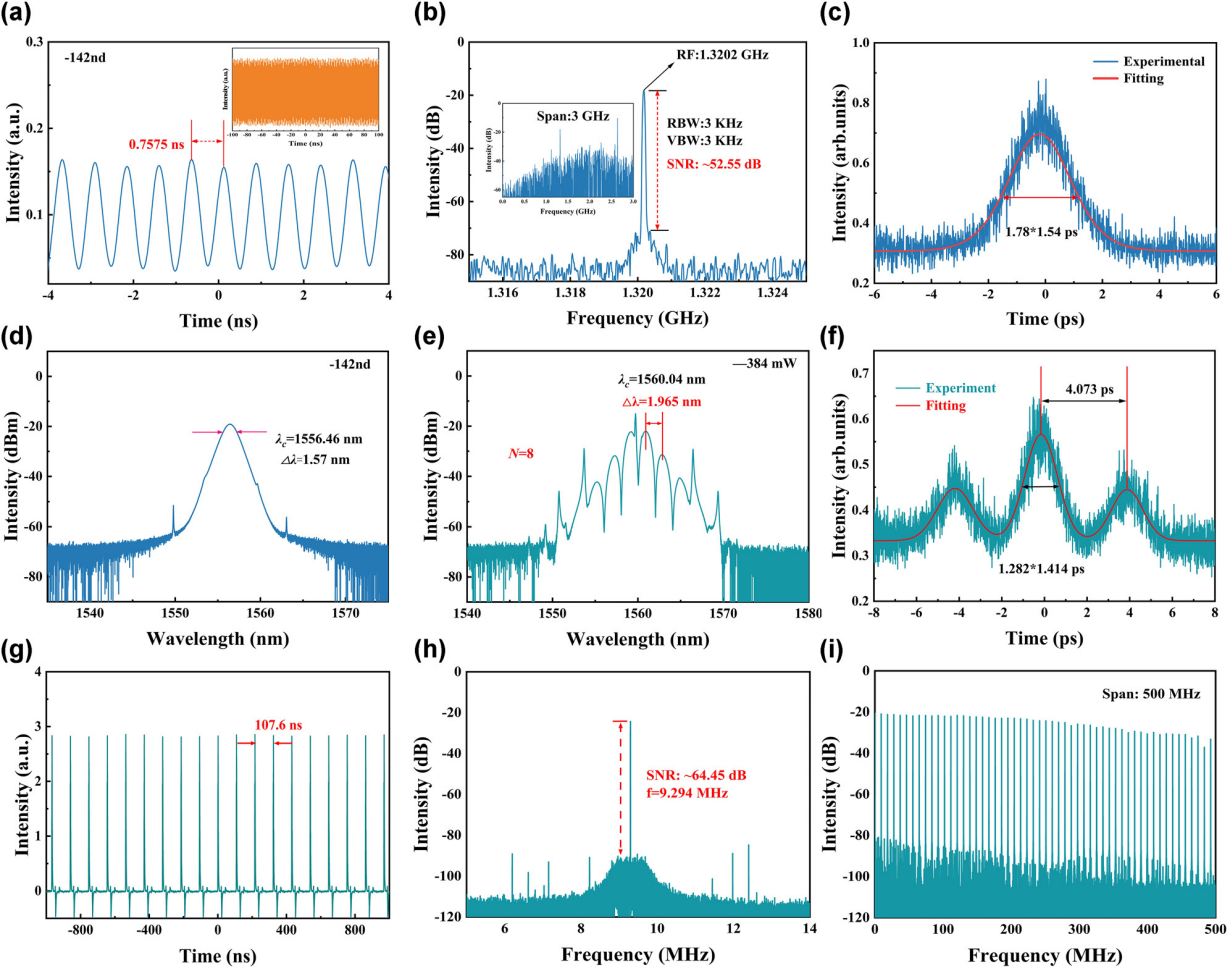
Characteristics of 142nd-order harmonic mode locking and bound-state solitons. (a) Pulse sequence of the 142nd-order soliton, (b) frequency spectrum of the 142nd-order soliton, (c) autocorrelation trace of the 142nd-order soliton, (d) spectrum of the 142nd-order soliton, (e) optical spectrum of bound-state solitons, (f) autocorrelation trace of bound-state solitons, (g) pulse sequence of bound-state solitons, (h) RF spectrum of bound-state solitons, and (i) RF spectrum of bound-state solitons with a 500 MHz sweep width.

At a pump power of 472 mW, a maximum repetition rate of 1.3202 GHz was achieved, corresponding to the 142nd-order HML. Figure 8(a)–(d) present the detailed characteristics of this high-order harmonic state. As shown in Figure 8(a), the measured pulse interval is 0.7575 ns, precisely 1/142 of the fundamental mode-locking interval. The inset of Figure 8(a) displays a long-duration pulse train, highlighting the excellent stability of the HML operation. The RF spectrum in Figure 8(b) further confirms this stability, with the measured repetition rate of 1.3202 GHz exactly matching 142 times the fundamental frequency, thereby validating the harmonic generation. Notably, the RF spectrum exhibits a high SNR of 52.55 dB, indicative of low noise and high spectral purity. Moreover, the inset of Figure 8(b), depicting a 3 GHz span of the RF spectrum, shows no spurious peaks, providing additional evidence of stable HML operation. The autocorrelation trace in Figure 8(c), fitted with a sech^2^ profile, yields a pulse duration of 1.78 ps. The corresponding TBP is calculated to be 0.346, suggesting slight chirping in the pulses. Spectral analysis in Figure 8(d) reveals a central wavelength of 1,556.46 nm and a 3 dB bandwidth of 1.57 nm. This spectral narrowing aligns with typical characteristics of high-order HML and further supports the soliton-like behavior of the pulses.

### 5.3 Bound-state solitons

A fiber laser operates as a dynamically balanced system, highly responsive to variations in cavity parameters such as dispersion, nonlinearity, gain, and loss properties. In a laser cavity with a net negative dispersion, the interplay between self-phase modulation and group velocity dispersion creates an environment conducive to the formation of optical solitons. As the pump power continues to rise, intensified nonlinear interactions combined with peak power limitations trigger the breakup of a single soliton into multiple subpulses, each exhibiting lower peak energy. These emerging pulses engage in complex interactions characterized by both repulsive and attractive forces. When these opposing forces achieve a dynamic balance, the system stabilizes, resulting in the formation of bound solitons with robust coherence [61], [62]. These are characterized by two or more pulses being bound together and moving as a group within the cavity, maintaining a constant time interval and fixed phase difference. In our study, a pair of stable bound solitons was observed when the pump power increased to 384 mW. Figure 8(e) shows the spectrum, which, in addition to the symmetric Kelly sidebands, exhibits modulation with a period of 1.965 nm. Furthermore, a significant dip at the central wavelength of 1,560.04 nm indicates that the phase difference between the bound solitons is *π*. The relationship between the spectral modulation period (Δ*λ*) and the time-domain pulse interval (Δ*τ*) can be expressed using the theoretical formula (Eq. (4)):

(4) $$\Delta \tau =\frac{\lambda_{c}^{2}}{c\Delta \lambda }$$

In this context, *λ*_*c*_ denotes the central wavelength of the bound solitons, while *c* represents the speed of light in a vacuum. According to this formula, the pulse interval Δ*τ* is calculated to be 4.128 ps. The autocorrelation trace shown in Figure 8(f) exhibits three distinct peaks, indicating the presence of a dual-pulse bound soliton mode-locking state. The peak spacing is measured to be 4.073 ps, which corresponds well with the theoretical soliton temporal interval Δ*τ*. Gaussian fitting reveals that the widths of the three peaks are approximately 1.282 ps, with an intensity ratio of 1:2:1, confirming that the dual solitons exhibit identical pulse durations and intensities. The time interval between adjacent solitons is 107.6 ns, corresponding to a repetition frequency of 9.294 MHz shown in Figure 8(g). The SNR of the bound-state soliton radio frequency spectrum recorded in Figure 8(h) is 64.45 dB, demonstrating high operational stability. Additionally, the spectrum within a 500 MHz range shown in Figure 8(i) further validates the soliton’s stability.

Table S2 summarizes the key performance parameters of previously reported systems alongside our work. Compared with prior studies, our laser demonstrates a significantly higher harmonic order of 142 and an ultra-high repetition rate of 1.3202 GHz, while maintaining a short pulse duration of 1.78 ps. These results confirm that our laser system not only achieves outstanding repetition performance but also preserves excellent pulse quality. Collectively, this highlights the effectiveness of the fabricated saturable absorber in enabling high-order harmonic mode-locking and its promising potential for high-speed optical communication and precision measurement applications. Building on this, Table 1 presents recent advancements in perovskite-based saturable absorbers for ultrafast lasers. Various perovskite materials have exhibited favorable nonlinear optical properties, supporting their application in mode-locked laser systems. For example, CsPbBr_3_ and CH_3_NH_3_PbI_3_ have demonstrated notable modulation depths and operational performance at near-infrared wavelengths, while Ba_2_LaTaO_6_ and PEA_2_PbI_4_ NCs have successfully extended operating wavelengths beyond 1,570 nm, enhancing their versatility in telecommunications and industrial laser applications. In this context, our work introduces BtzBiI_4_ as a distinctive lead-free alternative, offering both environmental sustainability and excellent nonlinear optical response. Its demonstrated ability to support high-order harmonic mode-locking at ultrahigh repetition rates distinguishes it from previously studied materials, underscoring its potential for demanding applications such as optical frequency comb generation and high-speed optical communication systems.

**Table 1:** Comparison of nonlinear optical properties and performance metrics of perovskite-based saturable absorbers.

Materials	MD	Pulse repetition rate (MHz)	*λ*_*c*_(nm)	Ref.
CsPbBr_3_	13.1 %	14.8	1,076	[63]
CH_3_NH_3_PbI_3_	8 %	4.08	1,064	[64]
CH_3_NH_3_PbI_3_	27.8 %	13.15	1,555	[65]
CH_3_NH_3_SnI_3_	15.2 %	4.03	1,064	[66]
(C_6_H_5_C_2_H_4_NH_3_)_2_PbI_4_	16 %	41.8	1,565.8/1,604	[67]
(PEA)_2_(CsPbBr_3_)_n−1_PbBr_4_	16.5 %	17.4	1,562/1,569.5/1,558.8	[68]
Ba_2_LaTaO_6_	24 %	12.3/550	1,557.4/1,556.6	[69]
PEA_2_PbI_4_ NCs	20 %	29.67	1,572.68	[70]
BtzBiI_4_	8.51 %	9.294/1,320.2	1,560.388/1,556.46	This work

## 6 Results and discussion

In this study, we successfully synthesized the bismuth-based perovskite crystal BtzBiI_4_ and fabricated a sandwich-structured BtzBiI_4_-SA, which was subsequently applied to an EDFL. The saturable absorption properties of BtzBiI_4_-SA were characterized using the balanced twin-detector technique, yielding a MD of 8.51 %, a *I*_sat_ of 47.19 MW/cm^2^, and a *T*_ns_ of 60.09 %. These results demonstrate its excellent saturable absorption performance. In an EDFL cavity with a total length of 22.11 m, fundamental mode-locking was achieved, producing pulses with a duration of 844 fs. By increasing the pump power and adjusting the polarization state, HML at orders of 28th, 41st, 55th, 65th, 70th, 76th, 92nd, 106th, 108th, 112nd, 114th, 122nd, 126th, 131th, and 142nd was observed, with the 142nd harmonic exhibiting a repetition rate as high as 1.3202 GHz. Additionally, bound-state soliton mode-locking was recorded at a central wavelength of 1,560.04 nm. The findings demonstrate that BtzBiI_4_ possesses outstanding nonlinear optical properties, positioning it as an excellent candidate for use as a SA in EDFLs. In addition to its impressive performance, BtzBiI_4_-SA exhibits remarkable environmental stability, cost efficiency, and a compact, alignment-free all-fiber design, highlighting its extensive potential across diverse applications. Particularly, the 1.3 GHz repetition rate pulses generated in this work fill a critical gap in the use of perovskite crystals as saturable absorbers in high repetition rate fiber laser systems. The laser’s high stability and narrow pulse characteristics make it well-suited for a wide range of applications, including optical communications, optical frequency combs, optical imaging, lidar, industrial laser processing, and nonlinear optics. This study paves the way for the broader adoption of BtzBiI_4_, a perovskite crystal, in ultrafast photonics, presenting promising avenues for future advancements in the field.

## Supplementary Material

Supplementary Material Details
