# Supplementary material for: High-repetition-rate ultrafast fiber lasers enabled by BtzBiI4: a novel bismuth-based perovskite nonlinear optical material
Source: Nanophotonics. 2025 Jun 30;14(15):2587–603. doi: 10.1515/nanoph-2025-0087 (PMC12322727; doi:10.1515/nanoph-2025-0087)
Supplement: Supplementary file 1 — Supplementary Material Details [file j_nanoph-2025-0087_suppl_001.docx]

Supplementary Material

Xiaohui Du, Chenyue Liu, Zefei Ding, Yuan Zhao, Cunguang Zhu*, Yaoyao Wang*, and Pengpeng Wang^[[1]](#footnote-1)^

High-Repetition-Rate Ultrafast Fiber Lasers Enabled by BtzBiI₄: A Novel bismuth-based Perovskite Nonlinear Optical Material

**
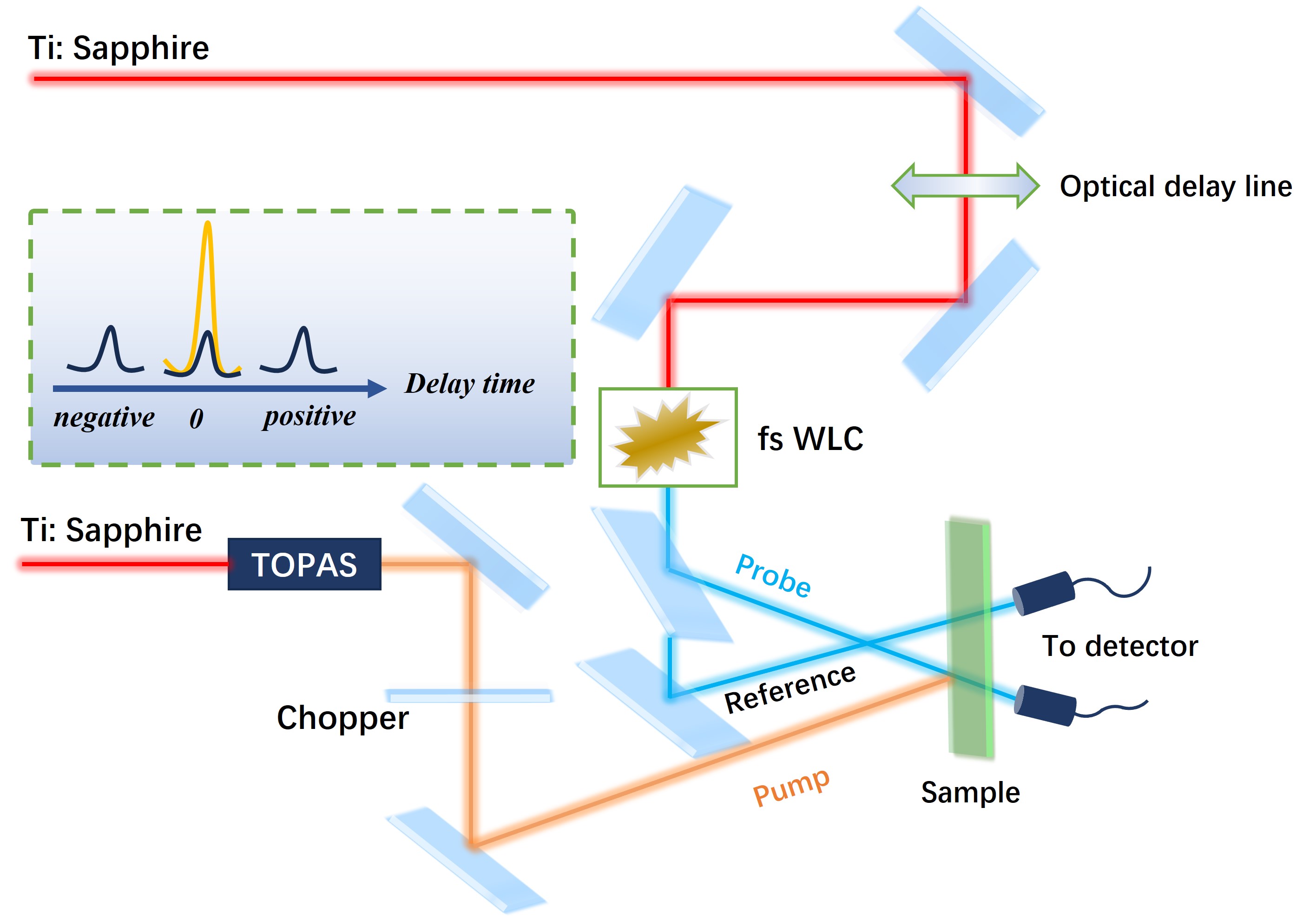
Table S1:** Comparison of Nonlinear Optical Parameters between BtzBiI_4_ and Conventional SAs.

| **SA** | **Modulation depth (Δ*T*)** | **Saturation intensity (*I*_sat_)** | **Ref*.*** |
| --- | --- | --- | --- |
| BP | 8.3% | 7.9 MW/cm^2^ | [1] |
| NbTe_2_ | 10.87% | 19.65 MW/cm^2^ | [2] |
| Ta_4_C_3_ | 23% | 0.127 MW/cm^2^ | [3] |
| Sb_2_Te_3_ | 36.16 % | 0.046 MW/cm^2^ | [4] |
| GeBi_4_Te_7_ | 6.6 % | - | [5] |
| InSe | 6.87% | 15.83 MW/cm^2^ | [6] |
| MXene-film | 1.22% | 0.4 MW/cm^2^ | [7] |
| MXene-DS | 3% | 0.2 MW/cm^2^ | [7] |
| BtzBiI_4_ | 8.51% | 47.19 MW/cm² | This work |

Figure.S1 Schematic optical layout of a typical ultrafast TA setup that features a tunable pump (delivered by a femtosecond optical parametric amplifier) and a broadband WLC probe (generated by focusing a small portion of the Ti:sapphire regenerative amplifier output in a transparent nonlinear crystal).[8]

The femtosecond transient absorption (fs TA) spectroscopy technique is a powerful method used to investigate ultrafast photophysical and photochemical processes, including excited-state dynamics, in materials. In our experiment [Figure.S1], the pump light (for sample excitation) is generated from the seed light (800 nm, 35 fs, 1 kHz, 6 mJ/pulse) via an optical parametric amplifier. After the sample is excited, another weak energy probe light, which is time-delayed with respect to the pump light and covers the white light spectrum, is used to measure the sample's absorption. The probe light is generated from the seed light through a white light crystal, and its weak energy ensures that it does not participate in the excitation of the sample. To obtain the absorption spectrum at different delay times, the optical path between the pump and probe lights is adjusted. After each delay time measurement, the absorption of the sample is calculated by differentiating the absorption measured by the probe light with respect to the baseline absorption before the pump light. This results in the absorption spectra at different wavelengths as a function of time. The wavelength is plotted on the x-axis, the delay time on the y-axis, and the absorption is represented by different colors to produce a false-color map. Additionally, part of the white light is directed through a separate optical path to the detector, which enhances the signal-to-noise ratio (SNR) of the collected data, as shown in Figure S1. The differential absorption is given by the following equation (Eq.1):

 (1)

△A(*λ*) represents the change in absorption at each wavelength, with the absorption of the probe light and reference light intensities denoted by I(*λ*)_pro_ and I(*λ*)_ref_, respectively. The ratio of absorption before and after the sample is pumped is expressed as [I(*λ*)_pro_/I(*λ*)_ref_]_unpump_ and [I(*λ*)_pro_/I(*λ*)_ref_]_pump_.

**Experimental Results and Discussion**

The spectral evolution, shown in **FigureS2. (a)**, remained stable, with only a slight shift of the central wavelength toward shorter wavelengths. Despite this shift, the 3 dB bandwidth showed minimal variation, reflecting robust spectral stability. Lastly, long-term stability tests further validate the robustness of the laser system. Continuous monitoring of pulse sequences on an oscilloscope over 12 hours, with hourly recordings [**FigureS2. (b)**], revealed consistent uniformity. Moreover, average output power fluctuations over 10 hours at a pump power of 365 mW remained within 7.9 mW [**FigureS2. (c)**]. This remarkable consistency highlights the high thermal damage resistance and excellent operational stability of the BtzBiI_4_-SA.

| 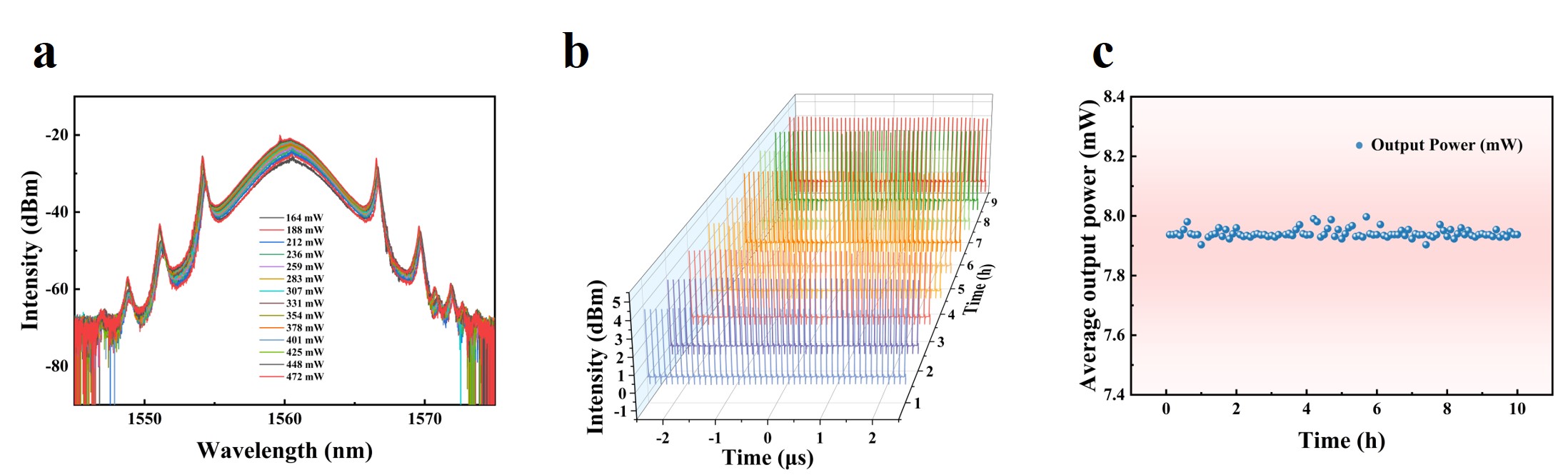 |
| --- |

**Figure.S2.** (a) Oscilloscope trace over 12 hours. (b) Changes in output power and single pulse energy with pump power. (c) Power stability.

| **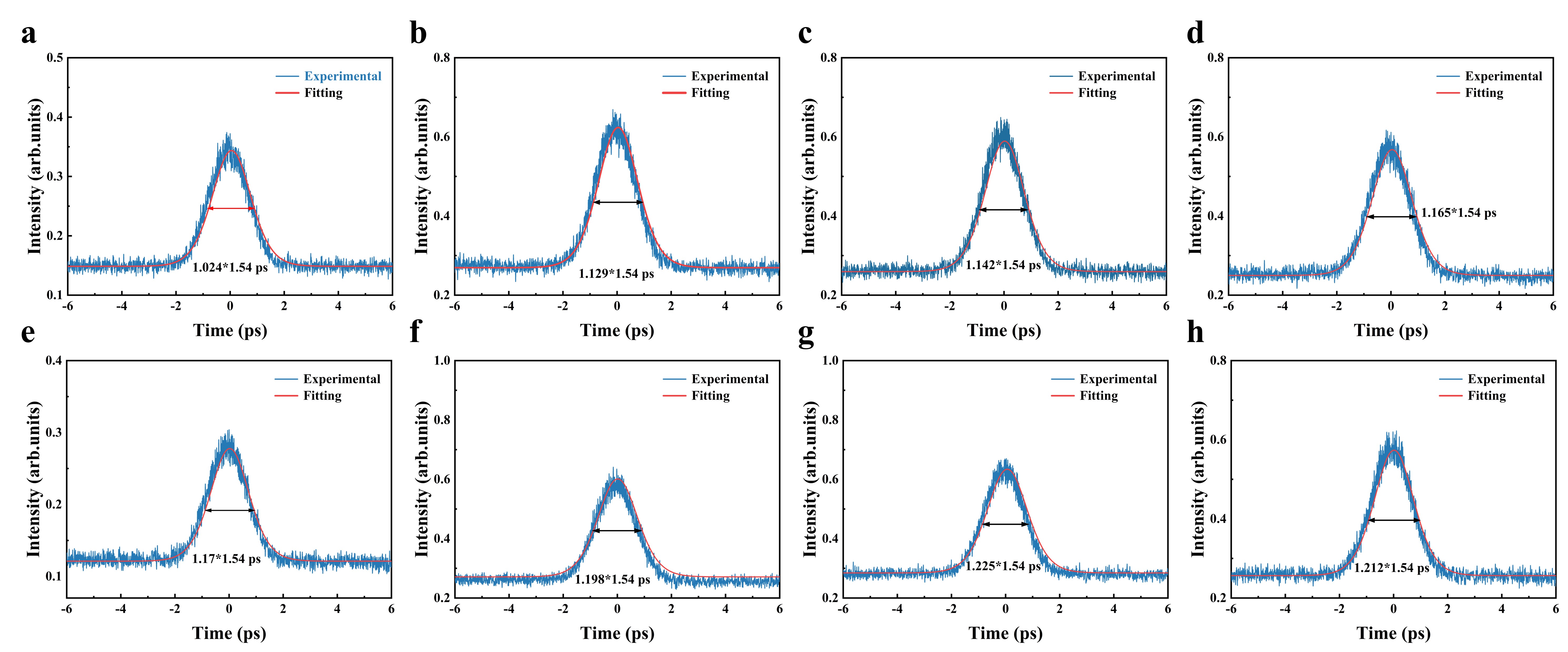** |
| --- |

**Figure.S3.** (a) 28th-order autocorrelation trace. (b) 41st-order autocorrelation trace. (c) 55th-order autocorrelation trace. (d) 65th-order autocorrelation trace. (e) 70th-order autocorrelation trace. (f) 76th-order autocorrelation trace. (g) 92nd-order autocorrelation trace. (h) 106th-order autocorrelation trace.

| 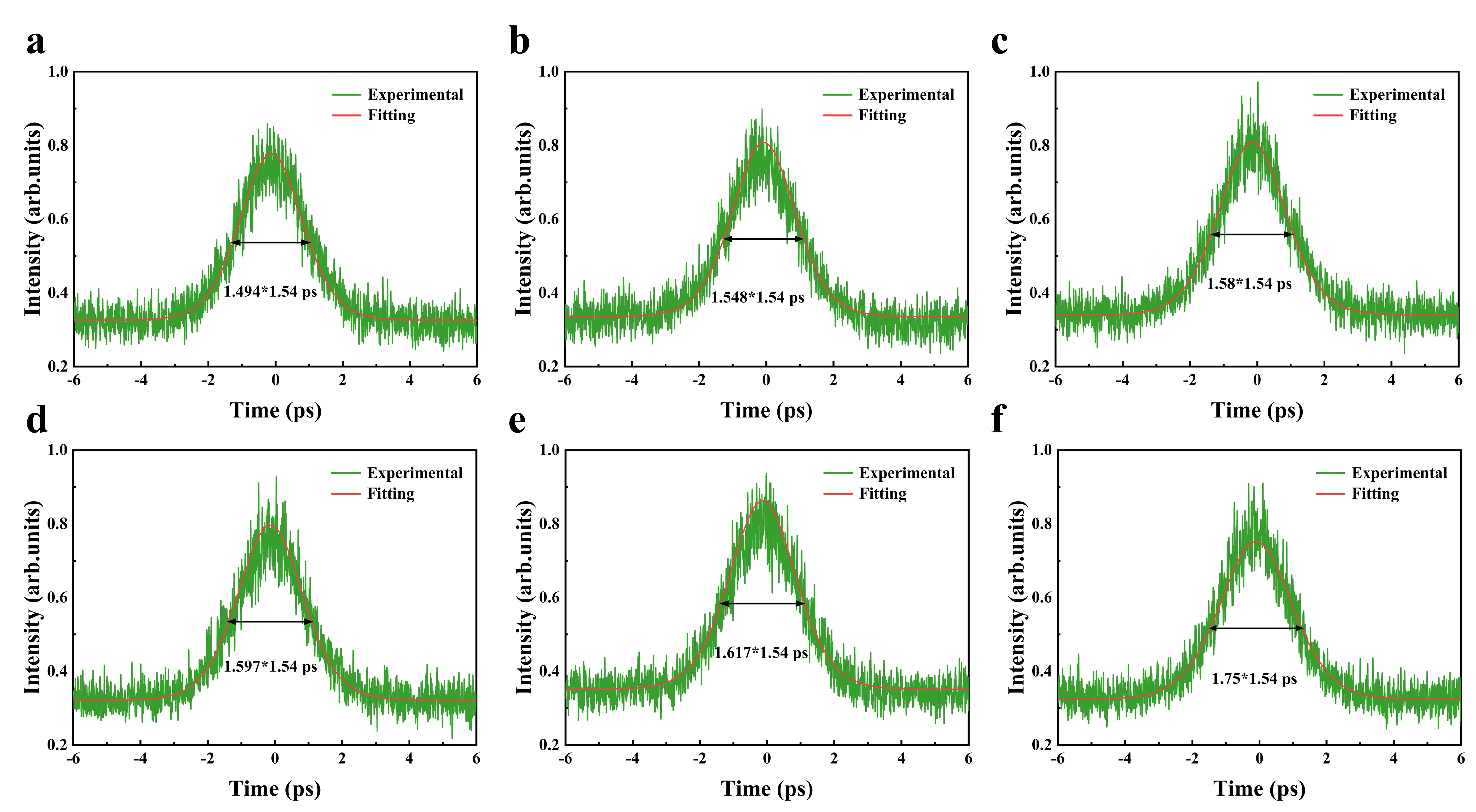 |
| --- |

**Figure.S4.** (a) 108th-order autocorrelation trace. (b) 112nd-order autocorrelation trace. (c) 114th-order autocorrelation trace. (d) 122nd-order autocorrelation trace. (e) 126th-order autocorrelation trace. (f) 131st-order autocorrelation trace.

**Table S2:** Recent studies on passively harmonic mode-locking fiber lasers.

| **Materials** | **Fundamental Frequency** | **Harmonic order** | **High repetition rate** | **Pulse duration** | **Ref.** |
| --- | --- | --- | --- | --- | --- |
| CNT | 12.8 MHz | 8th | 102.4 MHz | 1.6 ps | [9] |
| PbS | 8.5 MHz | 96th | 833 MHz | 981 fs | [10] |
| Cr_2_Si_2_Te_6_ | 12.6 MHz | 49th | 617.6 MHz | 3.2 ps | [11] |
| NG-VHS | 37.6 MHz | 36th | 1.205 GHz | 2.11 ps | [12] |
| MoS_2_/C | 13.12 MHz | 88th | 499.7 MHz | 2.91 ps | [13] |
| PbSnS_2_ | 4.342 MHz | 25th | 165.67 MHz | 1.235 ps | [14] |
| Pr-MOF | 3.99 MHz | 2nd | 7.98 MHz | 1.538 ps | [15] |
| EtbtBi_2_I_10_ | 4.86 MHz | 115th | 558.9 MHz | 1.706 ps | [16] |
| BtzBiI_4_ | 9.294 MHz | 142nd | 1.3202 GHz | 1.78 ps | This work |

**References**

[1] R. Zhao, et al., “Tunable high-power Q-switched fiber laser based on BP-PVA saturable absorber,” IEEE Journal of Selected Topics in Quantum Electronics, vol. 24, no. 3, pp. 1-5, 2017, DOI: 10.1109/JSTQE.2017.2714864.

[2] X. X. Shang, et al., “Niobium telluride absorber for a mode-locked vector soliton fiber laser,” Science China Physics, Mechanics & Astronomy, vol. 66, no. 5, p. 254211, 2023, https://doi.org/10.1007/s11433-022-2058-3.

[3] B. Gao, et al., “Ta_4_C_3_ MXene as a saturable absorber for femtosecond mode-locked fiber lasers,” Journal of Alloys and Compounds, vol. 900, p. 163529, 2022, https://doi.org/10.1016/j.jallcom.2021.163529.

[4] H. Ahmad, et al., “Thulium holmium-doped fiber laser mode-locked using Sb_2_Te_3_ saturable absorber coated arc-shaped fiber,” Infrared Physics & Technology, vol. 125, p. 104228, 2022, https://doi.org/10.1016/j.infrared.2022.104228.

[5] L. G. Guo, et al., “Tunable soliton molecules in mode-locked fiber laser based on GeBi_4_Te_7_ saturable absorber,” Optics & Laser Technology, vol. 157, pp. 108649, 2023, https://doi.org/10.1016/j.optlastec.2022.108649.

[6] W. Fan, et al., “Nanosized indium selenide saturable absorber for multiple solitons operation in Er^3+^-doped fiber laser,” Optics Express, vol. 31, no. 6, pp. 10176-10190, 2023, https://doi.org/10.1364/OE.484219

[7] A.A.A. Jafry, et al., “MXene Ti_3_C_2_T_x_ as a passive Q-switcher for erbium-doped fiber laser,” Optical Fiber Technology, vol. 58, p. 102289. <https://doi.org/10.1016/j.yofte.2020.102289>.

[8] Q. Zhang, Y. Luo, “Probing the ultrafast dynamics in nanomaterial complex systems by femtosecond transient absorption spectroscopy,” High Power Laser Science and Engineering, vol. 4, p. e22, 2016, https://doi.org/10.1017/hpl.2016.23.

[9] S. Yang, et al., “Widely-tunable harmonic mode-locked fiber laser by the combination of spectral filtering and gain management,” Optics & Laser Technology, vol. 157, p. 108726., 2023, https://doi.org/10.1016/j.optlastec.2022.108726.

[10] L. Nie, et al., “Harmonic Mode-Locked Er-Doped Fiber Laser Based on a Microfiber Based PbS Nanoparticle Saturable Absorber,” Particle & Particle Systems Characterization, vol. 40, no. 10, p. 2300075, 2023, https://doi.org/10.1002/ppsc.202300075.

[11] N. Xu, et al., “Low-threshold, multiple high-order harmonics fiber laser employing Cr_2_Si_2_Te_6_ saturable absorber,” Nanomaterials, vol. 13, no. 6, p. 1038, 2023, https://doi.org/10.3390/nano13061038.

[12] J. Chen, et al., “Two-dimensional conjugated metal-organic framework/graphene π–π stacked heterostructures for ultrafast photonics,” Advanced Functional Materials, vol. 34, no. 12, p. 2313027, 2024, https://doi.org/10.1002/adfm.202313027.

[13] S. Li, et al., “A multiple soliton state erbium-doped fiber laser based on a MoS_2_/C saturable absorber,” Journal of Materials Chemistry C, vol. 12, no. 42, pp. 17197-17205, 2024, DOI: 10.1039/D4TC02121E.

[14] X. Du, P. Wang, S. Li, C. Liu, Z. Ding, and C. Zhu, “Study on pulse characteristics of high-repetition-rate harmonic mode-locked laser utilizing PbSnS_2_ saturable absorber,” Infrared Phys Techn, vol. 137, p. 105179, 2024, <https://doi.org/10.1016/j.infrared.2024.105179>.

[15] X. Du, et al.., “Application of Pr-MOFs as saturable absorbers in ultrafast photonics,” J. Mater. Chem. C, vol. 12, no. 15, pp. 5400-5410, 2024, 10.1039/D4TC00340C.

[16] X. Du, et al., “Lead-Free EtbtBi_2_I_10_ Nanocrystals: a Promising Saturable Absorber for Ultrafast Photonics,” Advanced Optical Materials, 2403228. https://doi.org/10.1002/adom.202403228.

1. **Corresponding author:** **Fifth author**, Liaocheng University, Liaocheng, China; cunguang_zhu@163.com; 0000-0003-2343-7689 **Sixth author,** Liaocheng University, Liaocheng, China; wangyaoyao@lcu.edu.cn; 0000-0001-7562-6938

   **Seventh author**, Liaocheng University, Liaocheng, China; wangpengpeng@lcu.edu.cn; 0000-0002-0307-8837

   **First author:** Liaocheng University, Liaocheng, China; dd15169505459@163.com; 0009-0007-2705-9538

   **Second author:** Liaocheng University, Liaocheng, China; [13969147875@163.com](mailto:13969147875@163.com); 0009-0002-0323-9482 [↑](#footnote-ref-1)
